# Influencing Mechanism and Difference of Poultry Farmers’ Willingness and Behavior in Breeding Scale—Evidence from Jianghan Plain, China

**DOI:** 10.3390/ijerph19031631

**Published:** 2022-01-31

**Authors:** Yanqi Han, Hui Lyu, Shixiong Cheng, Yuhang He

**Affiliations:** School of Business, Hubei University, Wuhan 430062, China; hanyanqi321@hubu.edu.cn (Y.H.); 202021112011683@stu.hubu.edu.cn (H.L.); hyh@stu.hubu.edu.cn (Y.H.)

**Keywords:** poultry industry, breeding willingness, breeding behavior, impact mechanism, major public emergencies, Jianghan Plain

## Abstract

This paper uses the Heckprobit two-stage econometric model to explore the influence mechanism of poultry farmers’ willingness and behavior regarding scale based on 269 household survey data in the hinterland of Jianghan Plain, China. The results show that (1) family endowments, social capital, economic capital, product market prediction, and major public emergencies are the main influencing factors for farmers to engage in poultry farming; (2) economic capital, policy guarantees, product market prediction, and major public emergencies are the main factors that influence the changes in farmers’ poultry breeding scale; and (3) sampled poultry farmers are inconsistent between their breeding willingness and breeding behavior in poultry decision-making and the factors that affect the willingness and behavior are varied. Based on these findings, this paper suggests that the government should pay attention to inducing corresponding assistance and subsidy policies, formulating financial support countermeasures, organizing training and exchange meetings of the breeding industry, and promoting poultry market informatization to help the poultry industry prosper.

## 1. Introduction

The outbreak of the COVID-19 pandemic has seriously affected people’s lives and lifestyle. At the present stage, impact of COVID-19 on non-agricultural industry is much greater than that on agriculture, and the livestock and poultry industry is much more affected than the planting industry within agriculture [1]. Poultry products are an important part of the residents’ food basket of products in China. Since the outbreak of African swine fever in 2018, meat and egg poultry products have been selected as the main alternative products to pork, which is of great practical significance to ensure the nutrition and health of urban and rural residents at the table and the stable supply and price of agrifood. However, due to difficulties in the supply of feed and other materials and the hackneyed jam-up of agricultural products circulation, many poultry farmers have faced a survival crisis, resulting in short-term oversupply and falling prices of poultry products. Those further caused a problem resulting in the negative enthusiasm of poultry farmers to supplement fences, and some farmers face difficulties in resuming production due to severe economic losses, which brings big challenges to the poultry industry to restore its reproductive capacity and to guarantee the effective supply of poultry products in the later stage of the epidemic [2]. Nowadays, as COVID-19 continues, the scope of its impact on the poultry industry is becoming widened and deepened, which has a significant impact on the table supply of meat and egg poultry products for residents and the income level of poultry farmers. Therefore, it is necessary to study poultry farmers’ breeding intention and behavior mechanism to help the government department pushing the public health emergency governance mechanism, to improve the capability to cope with public health emergencies contingencies, and to provide data support and a policy basis for promoting the recovery and development of the poultry industry.

Since the outbreak of the COVID-19 pandemic, domestic and foreign scholars have conducted a large number of studies on the impact of COVID-19 on agriculture and the rural economy [3,4]. The research results include discussions on the impact of COVID-19 on specific industries, products, and links, such as food security [5], industrial production and development [6,7,8] (livestock and poultry, vegetables and fruits, dairy products, etc.), food international trade [9], and transportation [10], as well as reflections on agricultural risk management [11] and labor supply [12]. During the epidemic, poultry farms or households suffered great damage on the whole, with wide coverage of the variety of damage and a large number of damaged areas [13]. Present studies can mainly be divided into two types. The first type of study analyzes the impact of COVID-19 on livestock and poultry breeding. Zhu et al. [14] analyzed the impact of COVID-19 on the pig industry based on household survey data in 28 provinces and found that Chinese pig production and circulation were severely impacted in the short term, which slowed down the recovery of production capacity and increased the risk of pig price fluctuations. McEwan et al. [4] believes that Canada’s pork industry needs to focus on the bilateral pig and pork trade between Canada and the United States, the impact of potential absenteeism on the supply chain, and the global pig trade. Rude [15] used quarterly market models of cattle and beef markets in North America to examine the price and income effects associated with market disruptions and found that there was considerable price and income inhibition at all market levels. The second type of study analyzes the impact of COVID-19 on the poultry industry. Ye et al. [2] believed that the growth rate of poultry production is expected to decline significantly in 2020, caused by the pandemic, and that it may exacerbate the tight meat supply states for the reduced substitution effect of poultry consumption on pork consumption. Jiang et al. [16] also believed that the poultry industry would suffer great losses. However, some scholars believe that the breeding industry gradually phased out small and medium sized farms after the avian flu crisis in 2013; thus, the impact caused by the epidemic would be quickly compensated [17]. Weersink et al. [8] showed that the supply management sectors are more resilient to the impacts of COVID-19 than other sectors, as producers are generally more financially stable, losses are pooled, and production/marketing efforts are coordinated.

Poultry farmers’ decision-making and breeding strategy changes will induce changes in supply and demand in the poultry product market because they are the main participants and basic decision-making units in poultry farming. There are many factors that affect poultry farmers’ breeding and adjustment behavior. As the forerunner of breeding behavior, breeding willingness plays a guiding role in farmers’ actual decision-making behavior. According to cognitive psychology theory, people’s beliefs determine their preferences, which in turn further determine their behavior decisions. Moreover, poultry farmers who are willing to breed poultry at a particular scale are restricted by many factors, such as the endowment market, product market, policy, and economic environment [18], which may lead to gaps between final breeding behavior and breeding willingness. Research on farmers’ cognition and behavior decision-making includes factors such as the influence of perception, the influence of perception on behavior decision-making, the analysis of perception and adaptive behavior, and the consistency test between perception and behavior [19]. Specifically, in terms of agricultural fields, the research includes farmland circulation [20], ecological compensation [21], green production [22], and crop planting decision-making [23]. Few scholars have focused on the willingness and behavior of poultry farmers, and they analyzed the producers’ breeding intentions and behaviors of cleaner poultry production [24], breeding of laying hens [25], breeding of meat chickens [26], and breeding of mutton sheep [27]. Regarding the factors influencing poultry farmers’ willingness and behavior, relevant research shows that policies, individual characteristics of farmers, breeding characteristics, external environment awareness, and major public emergencies [28,29,30] will all have an impact on poultry production and development.

In summary, there are a lot of inspiring discussions about the impact of COVID-19 on the livestock and poultry industry and the countermeasures. However, previous studies need to be strengthened and supplemented in the following aspects. First, from the perspective of theme, with the sudden outbreak of COVID-19, scholars focused on governance mechanism construction and the crisis management of all walks of life in the face of public safety and health emergencies, and they put forward countermeasures to overcome the crisis. However, those studies have paid little attention to the issue of the influencing mechanism of poultry farmers’ willingness and behavior in the context of COVID-19. As the impact of COVID-19 continues, there is still a need to analyze the long-term impact of COVID-19 as well as the breeding willingness and the response of poultry farmers post-epidemic and in the post-epidemic era. Second, from the perspective of methodology, based on questionnaire data of micro farmers, previous studies have shown descriptive statistics and comparative analyses based on the impact of COVID-19 on livestock and poultry farms, whereas there is a lack of quantitative analysis of the impact of COVID-19 on poultry farmers by using quantitative models based on questionnaire data. Third, previous studies based on poultry farmers’ intention of breeding scale and breeding behavior is relatively insufficient. Farmers’ breeding intention has a fundamental impact on poultry farm size. However, systematic research on the intentions and behaviors of farmers as being relatively independent and interrelated factors is still lacking. Therefore, this research intended to conduct a supplementary study of the deficiencies of the previous literature regarding research themes, methods, and perspectives. This research constructed an analytic framework based on the theory of planned behavior, and then explored the main influencing factors and mechanism of the poultry farmers’ breeding willingness and behavior by using a Heckprobit two-stage model based on household survey data from Jianghan Plain in February 2021. Based on those findings, we provide decision-making reference for relevant government departments to make supportive subsidies for poultry farmers and promote the development of the poultry industry.

The rest of the paper is organized as follows. Section 2 presents the theoretical framework and data sources and econometric approach, as well as the data’s descriptive statistics, and it is followed by Section 3 that presents empirical results. Section 4 presents and discusses the empirical results and the future research prospect. Section 5 presents the conclusion, while Section 6 presents policy implications.

## 2. Materials and Methods

### 2.1. Theoretical Analysis of Influencing Factors for Farmers to Continue to Engage in Poultry Breeding

Based on the viewpoint of social cognitive theory [31], poultry farmers’ scale behavior is an interaction process of individual cognition, environment factors, and behavior itself. Specifically, farmers’ cognitions of poultry support policies and market prediction under the background of the COVID-19 epidemic are important aspects of analyzing individual cognition. Resource endowments, including social capital and economic capital, as well as the COVID-19 epidemic, are reflected in the environment factors to a certain extent. Farmers combine their cognitions and environmental factors based on their individual characteristics to produce specific poultry scale breeding willingness, although their willingness may be preliminary considerations based on an ideal state. If farmers choose to put breeding scale willingness into practice, they will take more consideration of the reality and will evaluate the behavioral risks, as well as being directly affected by individual cognition and environmental factors, after which the behavior of whether to adjust the breeding scale will be formed under the interaction of multiple factors.

**Figure 1:**
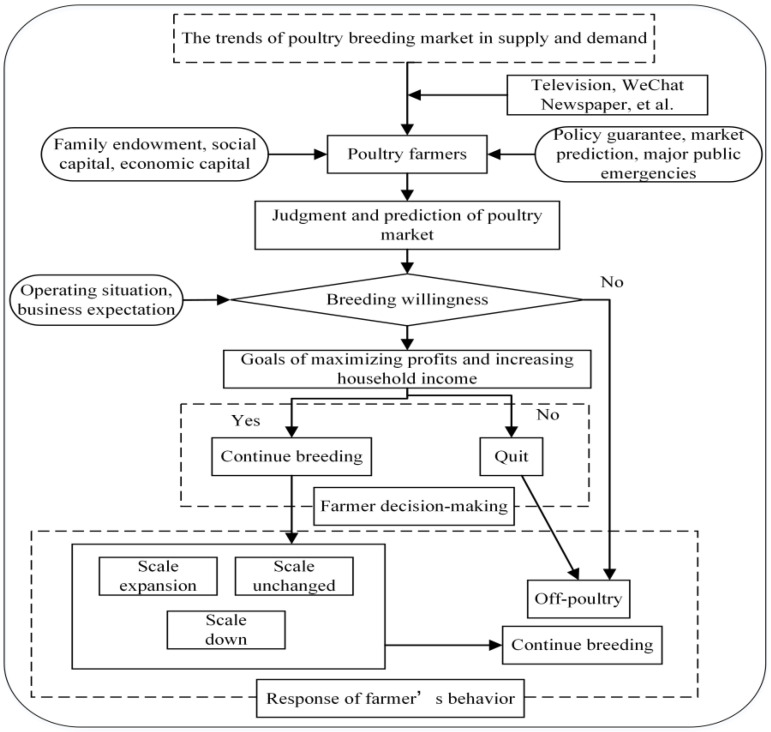
Mechanism framework of poultry farmers’ willingness and behavior.

Meanwhile, based on the theory of planned behavior, farmers’ willingness is the most direct factor affecting behavior [32]. Referring to previous studies [19,33], the theory of planned behavior also applies to the present study. The farmers’ breeding scale behavior is directly affected by breeding willingness. Farmers’ breeding willingness depends on the expectations of operating profit (behavior and attitude), prediction of the COVID-19 epidemic (subjective norms), and evaluation of the difficulty in realizing the breeding scale (perceived behavior control), which in turn affects breeding scale behavior. Under the assumptions of the rational economic man model, farmers’ breeding behavior decisions are affected by changes in expected profits; thus, farmers will choose a suitable breeding scale based on relevant support policies, environmental conditions, and individual characteristics. Generally, there may be three situations—namely, the breeding scale is expanded, it remains unchanged, or it is reduced. When the poultry farmers’ willingness to breed is weak, the farmers will leave the poultry industry and switch to other jobs (Figure 1).

### 2.2. Data and the Study Area

In this study, we used data drawn from a randomized field investigation of Chinese poultry farmers in Jingzhou, Tianmen, Xiantao, and Qianjiang, the hinterland of Jianghan Plain in Central China, from 19 to 28 February 2021, and the information refers to the production year of 2020. Farmers from a total of 85 townships in 11 counties and cities were interviewed face to face. Hubei Province has superior resources and conditions for developing high-quality poultry, and its poultry industry ranks at the forefront in China. Specifically, its egg poultry industry occupies an important position in the Hubei poultry industry. The Jianghan Plain is located in the south-central part of Hubei Province, named after the alluvial deposits of the Yangtze River and Han River. It starts from Zhijiang in Yichang in the west, reaches Wuhan in the east, Zhongxiang in Jingmen in the north, and connects with the Dongting Lake Plain in the south, covering an area of approximately 46,000 square kilometers. It is not only a land famous for fish and rice in China but also a region with an extremely developed livestock and poultry industry. The Jianghan Plain has always been a concentrated production area of pigs and poultry in Hubei Province and a key industrial area for high-quality poultry in Hubei Province (For details, please refer to the Construction Plan of Advantage Areas of Characteristic Agricultural Products in Hubei (2018–2022). The web link: http://farmigo.net/629 (accessed on 15 January 2022). The poultry production in Hubei Province was up to 532.45 million in 2018 from the data of Hubei statistical yearbook, and it ranked eleventh among 31 provinces. The eggs output in Hubei Province was 1.93 million tons in 2020, with a year-on-year growth rate of 8.10%, ranking sixth in China. Judging from the data of cities and states in Hubei province, the number of poultry cages was 382.90 million in 2020, while the number of poultry cages in Jianghan Plain, including the prefecture-level cities of Wuhan, Jingzhou, Xiaogan, Jingmen, and Yichang, was 221.35 million, accounting for 57.81% of the total number of poultry cages in 2020. Therefore, selecting this area as the research sample point has certain regional representativeness.

A multistage sampling strategy was adopted for selecting the respondents. In the first stage, eleven counties or cities were included: Jingzhou district, Shashi district, Jiangling district, Gong’an district, Jianli district, Shishou district, Honghu district, Songzhi district, Xiantao city, Qianjiang city, and Tianmen city. In the second stage, seven to eight townships within each selected county and city were randomly chosen, and eighty-five townships were chosen in total. In the third stage, two to four poultry farmers were randomly selected and interviewed in each township (Poultry farmers interviewed in this paper had a poultry breeding scale of 1000 or more. The breeding scale was divided into four grades: 1000–2000, 2000–5000, 5000–10,000, and over 10,000), resulting in a total of 269 respondents. Face-to-face interviews were conducted by well-trained census takers who worked at animal husbandry companies as salespersons, and the interviewees were their customers. The interviewers used a detailed, structured questionnaire. The survey gathered information covering household and farm-level characteristics (e.g., age, gender, education, household size, and farm size), farm poultry breeding production status, the use of production inputs (e.g., labor, funds, feed), the degree to which the poultry farmers were affected by COVID-19 (e.g., in their sales, demand, capital, credit), and poultry farmers’ breeding willingness and behavior. We computed the sample size using Cochran’s sample size determination equation due to a lack of information on the smallholder poultry farmer population in the sampled regions. Cochran’s equation is expressed as $N=Z^{2}\times P\times \left(1-P\right)/E^{2}$, where we assume a margin of error *e* of 5%, a probability or *p* value of 0.5, and a confidence level of 90% with a corresponding Z value of 1.64, thus yielding a minimum sample size of $N={\left(1.64\right)}^{2}\times \left(0.5\right)\times \left(0.5\right)/{\left(0.05\right)}^{2}$. Thus, collecting a random sample of at least 269 households was calculated to be enough to reach the confidence level required. Therefore, this study relied on a sample size of 269 respondents to ensure precision. All variables selected are detailed in Table 1.

### 2.3. Methodology

#### 2.3.1. Model Selection

To overcome the sample selection bias due to the fact that the poultry farmers’ change in behavior was a non-random sample of those farmers who were willing to continue farming, this study adopted the selection model for empirical analysis. Generally, the Heckman two-stage model can be used to estimate the problem of the dependent variable in the second stage being a continuous variable [34]. However, after the sample selection—that is, when the farmers hold the willingness to continue breeding—the dependent variable in the second stage is still a binary discrete explained variable, which needs to be processed by a binary selection model. Therefore, drawing upon the ideas of Van de Ven and Van Pragg [35] and Su and Wang [36], this study chose the sample selection binary probit model. This model uses the following selection equation: $Y_{i}^{*}=Z_{i}\gamma +\mu_{i}$, if $Y_{i}^{*}>0$, then $w_{i}=1$, otherwise $w_{i}=0$

(1) $$\mathrm{Prob}\left(w_{i}=1|Z_{i}\right)=\emptyset \left(Z_{i}\gamma \right)$$

(2) $$\mathrm{Result}\;\mathrm{equation}:\;(Y_{i}^{*}|w_{i}=1)=\beta X_{i}+\varepsilon_{i}\;\mathrm{And}\;\mathrm{match}\;\mathrm{the}\;\mathrm{conditions}:\;\mu_{i},\varepsilon_{i}\sim N^{2}\left(0,0;1,\sigma^{2};\rho \right)$$

where $W_{i}$ equal to 1 means that poultry farmers are willing to continue poultry farming, otherwise $W_{i}$ is equal to 0; where $X_{i}$ is the influencing factor of whether the breeding scale changes for poultry farmer i, $Y_{i}^{*}$ is the binary discrete dependent variable of breeding scale, and $Z_{i}$ is a vector of exogenous variables that determines the selection equation result. ∅  is a standard cumulative distribution function. The efficiency in the two-step estimation (Heckit) is inferior to the overall estimation of MLE because the error in the first stage is brought into the second stage. Thus, this study chose MLE to estimate. The likelihood function is as follows:

(3) $$\begin{array}{l} \ln L=\sum_{\begin{array}{l} i\in S\\ y_{i}\neq 0 \end{array}}w_{i}\ln\left\{\Phi_{2}\left(x_{i}\beta +offset_{i}^{\beta },z_{i}\gamma +offset_{i}^{\gamma },\rho \right)\right\}\\ +\sum_{\begin{array}{l} i\in S\\ y_{i}=0 \end{array}}w_{i}\ln\left\{\Phi_{2}\left(-x_{i}\beta +offset_{i}^{\beta },z_{i}\gamma +offset_{i}^{\gamma },-\rho \right)\right\}\\ +\sum_{i\notin S}w_{i}\ln\{1-\Phi \left(z_{i}\gamma +offset_{i}^{\gamma }\right)\} \end{array}$$

where *S* is an observable collection of $y_{i}$, $\Phi_{2}(\cdot )$ is the cumulative bivariate normal distribution function, Φ(·) is the standard cumulative normal distribution function, and $w_{i}$ is the optional weight of i. $\rho (\mu_{i},\varepsilon_{i})$ is the correlation coefficient of the error terms in the two stages. In the maximum likelihood estimation of Equation (3), ρ is not directly obtained by estimation, but atanh:

(4) atanhρ=1/2ln((1+ρ)/(1−ρ))

For Equation (4), if ρ is equal to zero, then atanh is equal to zero, and the binary probit based on sample selection will obtain the same result as the ordinary binary probit model. If ρ is not equal to zero, binary regression based on sample selection must be used to estimate the influence of the final independent variable on the dependent variable. Using a general probit model to process the result equation will produce biased estimation when ρ is not equal to zero. However, the Heckprobit estimation model provides a consistent and asymptotically effective estimation for all parameters of this type of model. Therefore, this study chose the Heckprobit two-stage model for regression.

#### 2.3.2. Variable Selection and Assignment

(1) Dependent variables. There are two types of dependent variables. One is the breeding willingness of poultry farmers. It was found that after the COVID-19 epidemic, 95.91% of the poultry farmers on the Jianghan Plain said they would continue to engage in the poultry industry, while 4.09% said they would not continue to engage in the poultry industry. Here, farmers’ willingness to continue to engage in the poultry industry can be divided into two categories: “No” assigned as 0 and “Yes” assigned as 1. The other category is whether the scale of poultry farmers’ breeding operations changed after this major public emergency. According to the survey, 3.35% of the farmers indicated that they would expand the poultry scale, while 32.71% of the farmers indicated that they would reduce the scale, but 63.94% of farmers would keep the current scale unchanged. In this study, we divided the situation of whether or not the farmers’ breeding scale changed into two categories. One category was that the breeding scale changed (including expanding or reducing), indicated by 1, while maintaining the breeding scale was indicated by 0.

(2) Independent variables. From the existing literature, the factors influencing farmers’ breeding willingness and behavior usually include personal characteristics, family characteristics, environmental characteristics, cognitive characteristics, livelihood capital, and policy support [27,33,37]. This study set up five types of independent variables that synthesized the general influencing factors in previous studies and that combined the research objectives: family endowment, social capital, economic capital, policy guarantees, and poultry market forecasts. In addition, according to the theory of planned behavior, farmers’ behavior should be viewed as farmers responding to external economic signals and making behavioral decisions based on external activities to maximize their own interests in a specific socioeconomic environment. Therefore, this kind of behavior is easily influenced by the social and economic environment [38,39,40]. Since the outbreak of COVID-19, its impact on the poultry industry has become an established fact [2]. Previous studies have shown that livestock and poultry farms or households are generally damaged, with a variety of types of damage and numerous damaged areas [13]. Therefore, this study set a major public emergency as the influencing factor to characterize the impact of a specific macroeconomic environment. (During the severe epidemic period from February to April 2020, epidemic prevention measures in China, such as village and road lockdowns, delayed the return to work at feed mills, suspended live poultry trading, closed slaughterhouses, etc. These delays and closures resulted in feed being unavailable for livestock and poultry in farms or households, the elimination of the young livestock and poultry trade, and the elimination of livestock and poultry slaughter at market, which made it difficult to carry out normal breeding and production activities, thus severely impacting livestock and poultry breeding. Compared with livestock breeding, the growth cycle of poultry breeding is generally short. According to the farmers interviewed, as far as the main breeds cultivated in Jianghan Plain are concerned, it takes approximately 45 to 55 days for table hen, 35 to 75 days for ducks, and 75 to 110 days for geese to be slaughtered, which generates a hurdle. What is more, the fresh-keeping period of poultry eggs is shorter, and the fragility is more obvious.) To fully reflect the impact of the COVID-19 epidemic as a major public emergency on livestock and poultry breeding, referential variables representing the COVID-19 epidemic and the prevention and control measures were selected, and the question items were set as follows: One was whether there were COVID-19-infected people or suspected patients in the village in 2020; next was how difficult it was for farms to maintain the movement of supplies and vehicles during the outbreak period in 2020; and third was the economic damage to farms caused by the epidemic. This research aimed to reflect the external environment and the progress of production with poultry farmers in major public emergencies and to measure their influence on poultry farmers’ willingness and behavior. The meanings and assignments of all variables are shown in Table 1.

### 2.4. Descriptive Statistics

The basic characteristics of the sample farmers are presented in Table 2. The table reveals that the survey subjects were dominated by men, accounting for 98.51%. Most of them were middle-aged, among which farmers aged 41–50 accounted for 55.75%, and percentage of farmers aged 51–60 was equal to those aged less than or equal to 40, accounting for 21.56% and 20.07%, respectively. The education level was mostly greater than or equal to junior high school, which accounted for 56.51% of the total sampled farmers. The farms for which there was no suspected or confirmed COVID-19 epidemic in the villages where the farmers were interviewed accounted for 82.53%. Most families were small families with 3–5 people, accounting for 66.17%. A total of 65.06% of families engaged in poultry breeding, accounting for 75–100% of the total family labor force. The main breeding type was egg poultry production, accounting for 84.76%. Regarding scale, the largest group of egg poultry breeding in the sampled regions was 2000–5000 feathers, accounting for 52.17%, and the second largest group was 5000–10,000 feathers, accounting for 33.04%. The proportions of farmers with a poultry breeding scale of 1000–2000 feathers and greater than 10,000 feathers were small.

## 3. Empirical Results

This study analyzed the influencing factors of poultry farmers’ willingness and behavior to continue poultry breeding (with changes in farming scale) in the context of the major COVID-19 public emergency with Stata13.0 software derived from college station (Stata, TX, USA) and using the Heckprobit two-stage model. In terms of independent variable selection, the set of independent variables in the second-stage result equation was a strict subset of the first-stage selection equation. After many analyses and verifications, in the second stage, the behavioral equation of farming scale was reduced to two variables, including poultry breeding type and age of household head. The Wald chi2 value of the model reached 69.09, and the value of Prob > chi2 was 0, indicating that the overall estimation effect of the model was better. The two-stage correlation likelihood ratio test of error terms showed that the P value was 0.0898, which passed the significance level test at 10% and rejected the original hypothesis, which means that the sampled data should suit the sample selection model. The estimated results are shown in Table 3.

### 3.1. Factors Affecting Farmers’ Willingness to Breed Poultry

Family endowment, social capital, economic capital, prediction of poultry breeding market, and major public emergency were the influencing factors for farmers to continue to engage in poultry farming. However, the policy guarantee was not significant. In terms of family endowment, the proportion of poultry breeding labor and the age of the household head had a significant positive impact on farmers’ willingness to continue poultry breeding, and they passed the 5% and 10% significance level tests, respectively. For social capital, e-commerce sales channels had a significant negative impact on farmers’ willingness to breed poultry and passed the 5% significance level test, which was not in line with expectations. The reason may be that traditional sales channels, such as distributors and retailers, are still the main sales channels of poultry products on the Jianghan Plain. In terms of economic capital, the pressure of repaying loans and the poultry breeding net profit in 2020 had significant positive and negative impacts on farmers’ willingness to breed poultry, and they passed the test of 10% and 1%, respectively. This shows that poultry farmers were more willing to continue farming under “less pressure for repaying loans” status and that when the loss of poultry breeding was lower, the willingness to continue poultry production was stronger. In terms of market forecasts, concern about the reduction in market demand for poultry products and the market prediction of the breeding industry had significant positive and negative impacts on farmers’ willingness to continue poultry production, and both passed the 5% significance level test. This shows that farmers who were less worried about the decrease in market demand for poultry products had a stronger willingness to continue poultry breeding, and the more optimistic farmers were about the breeding industry market, the stronger their willingness to continue poultry breeding was. The variable that represents the impact of major public emergencies, that is, whether there is COVID-19 in the village in 2020, had a significant negative impact on farmers’ willingness to continue poultry production and passed the 5% significance level test. This indicates that the COVID-19 epidemic infection or suspected cases in the village significantly reduced farmers’ willingness to breed poultry.

### 3.2. Factors Influencing Farmers’ Poultry Breeding Behavior

Economic capital, policy guarantees, poultry market prediction, and major public emergencies were the main influencing factors for poultry farmers to change the scale of poultry breeding operations. However, factors such as family endowment and social capital were not significant. In terms of economic capital, cash flow support time and net profit of the breeding industry in 2020 had significant negative and positive impacts on poultry breeding behavior, respectively. They were all significant at the 5% level, indicating that if poultry farmers had difficulty accessing credit or suffered from a large production loss in the poultry industry in 2020, they would be more likely to adjust their poultry breeding scale. In terms of policy guarantees, the relevant information provided by the government had a significant positive impact on poultry farming behavior and passed the 10% significance level test. This shows that poultry farmers who often received public service information about poultry market prediction, poultry market trends, and disease prevention and control from relevant government departments were more likely to adjust their breeding scale. Regarding market forecasts, the forecast of farming operating income had a significant positive impact on farming behavior, reaching a significance level of 1%. This shows that farmers who had less concern about breeding operational income were more likely to change the scale of their poultry breeding operation. As a variable representing the impact of major public emergencies, losses caused by the epidemic in 2020 had a significant positive impact on the change in farming scale, passing the 5% significance level test. This shows that poultry farmers were inclined to change their poultry scale when they suffered more serious economic losses caused by the epidemic in 2020.

### 3.3. Differences in Willingness and Behavior of Poultry Breeding Scale

Consistent with the theoretical expectation, this study found that poultry farmers often had inconsistent willingness and behaviors for various reasons. Table 4 shows that most households had consistent willingness and behaviors in poultry breeding, accounting for 95.91% or 258 households. Specifically, nine farmers were willing to continue poultry farming and expanded their scale of farming, and eighty farmers were willing to continue poultry farming and reduced their scale. They accounted for 9.28% and 82.47% of the farmers who changed their farming scale, respectively. A total of 33.09% of the total sample of farmers were willing to continue in the poultry industry and changed their farming scale. However, there were 169 farmers who were willing to continue farming but had not changed the scale of farming operations at all, accounting for 62.83% of the total sampled farmers. According to our survey, the main reasons for differences between farmers’ willingness and behavior to continue poultry breeding included frequent shortage of poultry operation capital, market decline in the poultry farming industry, and changes in decision-making planning regarding the family labor force going out to work. In addition, according to the results, the influencing factors of farmers’ willingness and behavior to continue poultry breeding were generally quite different in direction and intensity.

Poultry breeding aims to achieve stable production and supply of poultry products. Generally, previous studies have shown that improving the prices of the factor market and the price of the poultry product market promotes market informationization [1] to realize the unity of poultry farmers’ willingness and behaviors. In addition, according to the investigation, to promote the agreement of poultry farmers’ willingness and behavior, it is necessary to provide subsidy policies for poultry breeding, financial policy support, and measures to eliminate farmers’ panic. Among them, special policy support from relevant departments and credit support from banks and other financial institutions can help farmers in the long term and short term, respectively, overcome poultry breeding difficulties, solve the funding gap of breeding industry operation, and restore the reproduction capacity of the breeding industry. After the outbreak of COVID-19, more attention should be given to investing resources to mobilize market exchange meetings and develop prospects of livestock and poultry breeding industries, to help farmers understand the market supply and demand trend, and to develop a plan for the poultry breeding industry, as well as to eliminate panic and boost confidence among farmers in continuing their poultry breeding.

## 4. Discussion

Previous studies focused on farmers’ breeding behavior based on the theory of planned behavior. Internal and external control factors are the main influence factors of farmers’ breeding behavior. The internal control factors include the individual characteristics of a farmer’s age, gender, education level, etc., while the external control factors include production characteristics, environmental characteristics, and risk perception characteristics. In the present study, family endowment, social capital, economic capital, policy guarantee, market prediction, and major public emergencies were set up to analyze the impact of them on the poultry farmers’ breeding intentions and breeding behavior. Wu [18] et al. (2019) believed that the age of household head had no impact on the breeding behavior, but had a significant negative impact on breeding willingness, while the number of farming labors had a significant positive impact on both breeding willingness and breeding behavior. The conclusion consistent with the present study is that family endowment has an impact on breeding willingness, while the difference is whether family endowment has an impact on breeding behavior. In terms of economic capital, Yang [41] et al. (2011) believed that the difficulty of obtaining funds was positively correlated with farmers’ willingness to raise pigs, and the finding was significant at the 5% level when analyzing the factors influencing farmers’ willingness to raise pigs. The more difficult it was to obtain funds, the higher the farmers’ willingness to raise pigs. This is consistent with the conclusion that the breeding net profit in 2020 set in this study had an impact on poultry breeding willingness. That is, the net profit of breeding at the end of the year was negatively correlated with farmers’ willingness to raise pigs. The lower the net profit at the end of the year was, the higher farmers’ willingness to raise pigs was. This may be related to the fact that poor farmers desire to broaden income channels and improve household economic conditions through sideline activities such as breeding.

From the difference in the influencing mechanism between the willingness and scale of farming behavior, the influencing factors of the willingness and scale of farming behavior were quite different in influencing direction and intensity. Among those factors, only a small number of factors showed certain consistency in the direction of influence, while there were still differences in intensity. For example, in terms of economic capital, the lower the reduction in the net profit of farming in 2020 compared with 2019, the stronger the farmers’ willingness to breed and the higher proportion of adjustment of the scale of farming, but the influencing intensity definitely varied. Family endowment and social capital only had a significant impact on poultry breeding willingness, while policy guarantee only had a significant impact on the behavior of scale farming adjustment. In terms of the market prediction, the influence on farmers’ willingness to breed was reflected in product demand prediction and poultry market prediction, while the impact on farmers’ behavior of adjusting farming scale was reflected in the prediction of breeding operation income. In the aspect of major public emergencies, the impact on farmers’ willingness to breed was reflected in whether there was a COVID-19 epidemic in the village, while the impact on farmers’ behavior of scale adjustment was reflected in the amount of economic damage caused by the COVID-19 epidemic. To a certain extent, this suggests that although farmers’ cognition can play a role in farmers’ behavior decision-making, in reality, it is mostly influenced by some external environmental factors and farmers’ personal characteristics, while there may be some accidental factors as well.

This study also had some limitations that should be mentioned. First, the sample size was relatively small in this study, and the survey scope of sample data was too small to be convincing. Second, the present study only covered meat and egg poultry products, and put forward countermeasures and suggestions for the development of meat and egg poultry products. Last, in view of the differences in production risks and dilemmas, as well as the ability to adjust production behaviors faced by farmers of different scales under public security emergencies, the present paper did not distinguish the heterogeneity of the poultry farmers’ willingness and breeding scale adjustment behavior.

Further studies should be carried out when more data are available. First, a future study will expand the sample size and scope to make the results more convincing. Second, a future study will include more poultry species, making the coverage of poultry species more comprehensive, thus resulting in the ability to put forward countermeasures and suggestions for the development of the poultry industry from the perspective of industry. Last, a future study will consider separating the groups according to their size (for example, large vs. small-medium size) to show which factors affect more the willingness and behavior of one group with respect to another.

## 5. Conclusions

In this paper, we theoretically and empirically analyzed the influencing mechanism of poultry farmers’ willingness and behavior by employing a Heckprobit two-stage model and household survey data collected from 269 poultry farmers in the hinterland of Jianghan Plain in rural China. The main conclusions are as follows:

(1) Family endowment, social capital, economic capital, prediction of breeding market, and major public emergencies are all influencing factors for poultry farmers to continue to engage in poultry breeding, but policy guarantees have no significant impact on farmers’ willingness to breed. 

(2) Economic capital, policy guarantees, prejudgment of the breeding market, and major public emergencies are the influencing factors for poultry farmers to change the scale of breeding operations, but the influence of family endowment and social capital are not significant.

(3) The sampled poultry farmers showed inconsistent breeding willingness and behaviors, and there was a large difference between the factors affecting the willingness and behaviors of poultry farmers.

## 6. Policy Implications

Our findings in this study have important policy implications for healthy poultry production and industry development. First, relevant local government departments should introduce corresponding assistance policies and subsidy mechanisms, paying special attention to assisting free-range poultry farmers in overcoming financial difficulties, boosting confidence of the poultry farmers in breeding behavior, and promoting poultry farmers to increase their income to help the development of poultry breeding markets. Next, banks and other financial institutions should be encouraged to implement financial policies for the steady growth of the poultry industry. Free-range poultry farmers can be provided with preferential short-term liquidity support to alleviate capital turnover difficulty in the short term, while special refinancing for poultry breeding can be provided to guide financial institutions in improving the efficiency of financial services and the availability of financial services to free-range households and to enhance their breeding reproduction ability in the long term. Then, more attention should be given to investing certain resources to mobilize and organize exchange meetings on the market status and development prospects of the livestock and poultry breeding industry when major public emergencies outbreak, which would help farmers respond to changes in market information in a timely and accurate manner and reduce the lag and blind obedience of farmers’ production decisions. Finally, the information development of the poultry industry should be promoted. We can achieve this through the market-oriented development of poultry breeding information to narrow the information asymmetry between the factor market and the product market of the poultry industry and to decrease the market risks faced by farmers, as well as to improve the unity of the willingness and behavior of poultry production.

## Figures and Tables

**Table 1:** Definition and descriptive statistics.

Variable Category	Variable Name	Meaning and Assignment of Variables	Mean	SD
Dependent variable				
	Poultry breeding behavior	Past behavior; 1 if the poultry production scale changed, 0 otherwise	0.3606	0.4811
	Poultry breeding willingness	1 if farmers continue to engage in poultry industry, 0 otherwise	0.9591	0.1984
Independent variable				
Family characteristics	Proportion of poultry farming labor	The proportion of poultry farmers to total labor force (%), and the value is 0–100	81.8711	26.5031
	Total population	Total population in the family	4.1004	1.3219
	Poultry type	1 if the type is hatch farm, 2 if egg poultry farm	1.8476	0.3601
	Age	Age of household head (years)	46.0781	6.6522
Social capital	E-commerce platform	1 if an e-commerce sales channel exists, 0 otherwise	0.0446	0.2068
	Access to credit	1 if household has access to credit, 0 otherwise	0.1636	0.3706
Economic capital	Repayment pressure	Repayment pressure at poultry operation is very large = 1, larger = 2, average = 3, smaller = 4, very small = 5	2.1599	1.2873
	Duration of cash flow	The cash flow can support 7 days = 1, 7–15 days = 2, 15–30 days = 3, over 30 days = 4	3.0223	0.7819
	Poultry production net profit in 2020	Unchanged compared with last year = 1, 30% less than last year = 2, 30–50% less than last year = 3, reduced by more than 50% compared with last year = 4	2.3606	0.617
Policy guarantee	Government information	1 if household received information from local government departments, 0 otherwise	0.052	0.2225
	Financial support policy for poultry industry	The types of financial support policies, including special refinance to breeding industry, credit with preferential interest rate and financial discount support, improved loan repayment margin, and increased insurance claim rate and financial service efficiency	2.6691	1.0747
Market forecast	Prediction of income	The degree of worry about the reduction in operational income, sorted from high to low as 1, 2, 3, 4, and 5.	1.2639	0.5739
	Prediction of demand	The degree of worry about the decrease in market demand, sorted from high to low as 1, 2, 3, 4, and 5.	1.5985	1.0414
	Market prediction	The prediction of poultry production demand market, sorted from optimistic to pessimistic as 1, 2, 3, 4, and 5.	3.0855	0.5828
Major public emergency	Was there any epidemic in village in 2020?	1 if people in the village are infected or suspected of being infected by COVID-19, 0 otherwise	0.1747	0.3804
	Lockdown level from Feb to Apr in 2020	Materials and traffic can go in and out, and the procedures are relatively simple and easy = 1; can go in and out, and the procedures are generally complex = 2; can go in and out, but the procedures are very complicated = 3; materials and vehicles are almost inaccessible = 4	3.2305	0.616
	Damage caused by COVID-19 in 2020	<CNY 10,000 ‡ = 1; CNY 10,000–30,000 = 2; CNY 30,000–50,000 = 3; CNY 50,000–100,000 = 4; >CNY 100,000 = 5	2.7026	1.0724

Note: ‡ CNY (yuan) is Chinese currency: USD 1 = CNY 6.44 in 2021. SD, standard deviation.

**Table 2:** The sample’s basic characteristics.

Variable	Category	Households Number (Households)	Proportion (%)	Variable	Category	Households Number (Households)	Proportion (%)
Gender	Man	265	98.51	Family size (person)	≤2	81	30.11
	Woman	4	1.49		3~5	178	66.17
Age	≤40	54	20.07		≥6	10	3.72
	41~50	150	55.76	Labor proportion in poultry breeding (%)	≤25	20	7.43
	51~60	58	21.56		25~50	47	17.47
	61~70	7	2.60		50~75	27	10.04
Education	Below primary school	81	30.11		75~100	175	65.06
	Junior high school dropout	36	13.38	Poultry breeding type	Hatchery	41	15.24
	Junior high school	101	37.55		Eggs breeding	228	84.76
	High school (technical secondary school)	41	15.24	Poultry breeding scale (feather)	1000~2000	16	6.96
	College	10	3.72		2000~5000	120	52.17
COVID-19	Yes	47	17.47		5000~10,000	76	33.04
	No	222	82.53		≥10,000	18	7.83

**Table 3:** Regression results of Heckprobit two-stage model.

Influencing Factor		Willingness to Continue Farming in the First Stage			The Second Stage of the Scale Change (Behavior)		
Category	Variable	Coefficient	Standard Error	Z-Value	Coefficient	Standard Error	Z-Value
Family endowment	Proportion of poultry breeding labor	0.0218 **	0.0099	2.2	0.0001	0.0048	0.01
	Total family population	0.2543	0.2818	0.9	0.0949	0.1021	0.93
	Poultry breeding type	−5.3747	0.205	0	—	—	—
	Age of household head	0.0650 *	0.0335	1.94	—	—	—
Social capital	E-commerce sales channel	−1.5956 **	0.7899	−2.02	0.7618	0.554	1.38
	Access to credit	−0.1566	0.5357	−0.29	−0.1511	0.2585	−0.58
Economic capital	Repayment pressure at poultry industry	0.4928 *	0.271	−1.82	0.0115	0.0935	0.12
	Support time of cash flow in poultry breeding	0.1507	0.2542	0.59	−0.3087 **	0.1471	−2.1
	Net profit of poultry breeding in 2020	−0.3554 ***	0.3973	−0.89	0.3661 **	0.1827	2
Policy guarantee	Whether the government provides breeding information	−0.0063	1.342	0	0.7591 *	0.3981	1.91
	Financial institution support policy	−0.3855	0.3437	−1.12	0.1611	0.1088	1.48
Market forecast	Pre-judgment of poultry industry operating income	0.7716	0.5956	1.3	0.6740 ***	0.2268	2.97
	Concern with degree of reduction in poultry products market demand	0.6485 **	0.3088	2.1	−0.1875	0.1333	−1.41
	Prediction of poultry breeding market	−1.0300 **	0.4518	−2.28	0.17489	0.1699	1.03
Major public emergency	Whether had a COVID-19 outbreak in village in 2020	−1.3776 **	0.695	−1.98	0.0888	0.3313	0.27
	Difficulty of the village’s supply vehicles entering and leaving from February to April 2020	−0.4026	0.6781	−0.59	0.3617	0.1803	−2.01
	Damage caused by COVID-19 in 2020	0.1631	0.3186	0.51	0.1386 **	0.1108	1.25
	_cons	12.3836	0.41	0	−1.5269	1.3264	−1.15
LR test of independent equations (rho = 0): chi2(1) = 2.88 Prob > chi2 = 0.0898							
Log likelihood = −148.8315							
Wald chi2(15) = 69.09							
Prob > chi2 = 0.0000							

Note: ***, **, and * indicate that values significant at the 0.01, 0.05, and 0.1 level, respectively.

**Table 4:** Poultry farmers’ willingness and scale change status.

	With Actions to Change the Poultry Breeding Scale						Without Poultry Farming Scale Change Action	
	Scale-Up/Households	Proportion/%	Scale-Down/Households	Proportion/%	Poultry Scale Changed/Households	Total/%	Households	Proportion/%
With willingness to continue farming poultry breeding	9	9.28	80	82.47	89	33.09	169	62.83
Without willingness to continue poultry breeding	0	0	8	8.25	8	2.97	3	1.12

## Data Availability

The data presented in this study are available on request from the corresponding author.
